# A Bibliometric and Visualized Analysis of Cardiac Regeneration Over a 20-Year Period

**DOI:** 10.3389/fcvm.2021.789503

**Published:** 2021-12-13

**Authors:** Siyuan Ma, Junyu Yan, Lu Chen, Yingqi Zhu, Kaitong Chen, Cankun Zheng, Mengjia Shen, Yulin Liao

**Affiliations:** State Key Laboratory of Organ Failure Research, Guangdong Provincial Key Laboratory of Shock and Microcirculation, Department of Cardiology, National Clinical Research Center of Kidney Disease, Guangdong Provincial Institute of Nephrology, Nanfang Hospital, Southern Medical University, Guangzhou, China

**Keywords:** cardiac regeneration, bibliometric analysis, cardiomyocyte proliferation, induced pluripotent stem cell, extracellular vesicles, direct cardiac reprogramming, macrophages, microRNAs

## Abstract

**Background:** Recent research has suggested that cardiac regeneration may have the widely applicable potential of treating heart failure (HF). A comprehensive understanding of the development status of this field is conducive to its development. However, no bibliometric analysis has summarized this field properly. We aimed to analyze cardiac regeneration-related literature over 20 years and provide valuable insights.

**Methods:** Publications were collected from the Web of Science Core Collection (WoSCC). Microsoft Excel, VOSviewer, CiteSpace, and alluvial generator were used to analyze and present the data.

**Results:** The collected 11,700 publications showed an annually increasing trend. The United States and Harvard University were the leading force among all the countries and institutions. The majority of articles were published in Circulation Research, and Circulation was the most co-cited journal. According to co-citation analysis, burst detection and alluvial flow map, cardiomyocyte proliferation, stem cells, such as first-and second-generation, extracellular vesicles especially exosomes, direct cardiac reprogramming, macrophages, microRNAs, and inflammation have become more and more popular recently.

**Conclusions:** Cardiac regeneration remains a research hotspot and develops rapidly. How to modify cardiac regeneration endogenously and exogenously may still be the hotspot in the future and should be discussed more deeply.

## Introduction

Heart failure (HF) is a leading cause of death worldwide, and effective treatment methods are urgently needed. Although the existing treatments of HF, such as drugs and cardiac resynchronization therapy, are widely applied in clinical practice, they cannot restore the normal number of cardiomyocytes (CMs) and cardiac function (1, 2). One of the most important reasons is that CMs lose the capacity to proliferate in adulthood (3). Current studies have found that human CMs have the ability to self-renew, while the rate is only approximately 0.3–1% per year and too low to restore normal cardiac structure and function (3). Increasing the proportion of normal functional CMs is the primary method for recovering cardiac structure and function after a myocardial injury. Heart transplantation may be the best and last choice but is restricted by the small number of donors, high costs, and surgical complexities. Therefore, cardiac regeneration is necessary (4).

With the emergence of the idea of cardiac regeneration, many methods for cardiac regeneration are being explored, such as cell-based therapy using induced pluripotent stem cells (iPSCs) and gene therapy by artificially mediating cell cycle-related factors (5, 6). However, to date, no effective approaches are available to regenerate the damaged human heart due to obvious limitations in clinical application: lower engraftment rate, clinical complications after transplantation, and unbalanced electromechanical conduction between the transplanted cells and resident cells (7–9). Therefore, the main task of researchers is to optimize the existing schemes or find a new scheme. To obtain the most valuable content and promote development in the field of cardiac regeneration, an in-depth understanding of the current status is necessary.

**Figure 1:**
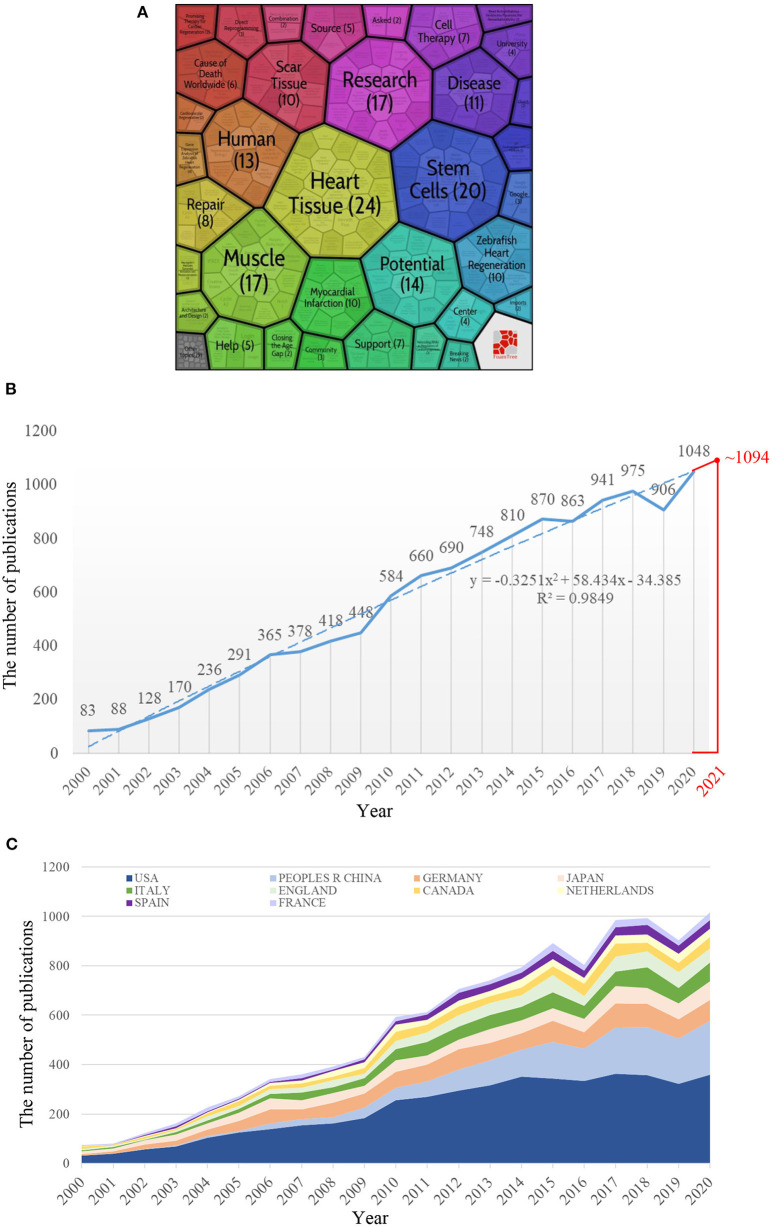
The annual number of publications worldwide or top 10 productive countries/regions. **(A)** The first 100 publications of a web search. **(B)** The annual number of publications worldwide from 2000 to 2020. The fitting formula was y = −0.3251x^2^ + 58.434x – 34.385. **(C)** The annual number of publications of the top 10 productive countries/regions from 2000 to 2020. Among the 10 countries, the People's Republic of China has seen the fastest growth in the number of publications in recent years.

In this study, we used Carrot 2, a document clustering tool (https://search.carrot2.org/#/search/web), to initially form trees about cardiac regeneration based on the first 100 publications of a web search (Figure 1A). The results only hinted that heart tissue (10), research (11), and muscle (11) were the top three leading topics, which was not enough for us to accurately understand the developmental status of cardiac regeneration. Establishing a more comprehensive understanding of the field of cardiac regeneration is paramount. A bibliometric analysis is a quantitative and qualitative method that uses a variety of software systems to evaluate the scientific output and research trends in a certain research field, which greatly reduces the risk of manual bias in the analysis (12). In contrast, conventional reviews require a manual selection of articles and are prone to missing key information. To our knowledge, no bibliometric analysis of cardiac regeneration has been performed. Our aim was to use this method to examine the current status and emerging trends in cardiac regeneration-related research, to provide an in-depth evaluation of its developmental status for the knowledge of researchers, and to guide their future work.

## Methods

### Search Strategies

The publications were collected from the Web of Science Core Collection (WoSCC). The search terms were used as follows: TS = (cardiac regenerat^*^) OR (heart regenerat^*^). Only articles and reviews written in English were included. The purpose of this study was to provide an overview of the development of cardiac regeneration in the twenty-first century, and we did find that the number and annual increment of publication associated with cardiac regeneration prior to the year 2000 were low (Supplementary Table 1). Therefore, we chose the year 2000 as the starting point for the retrieval year. Also, we chose 2020 as the end point of the retrieval year to analyze all the publications in each year.

### Data Collection and Analysis

All data to be studied, such as the annual number of publications, and the number of articles published by countries/regions, institutions, funding agencies, journals, and authors were downloaded as plain text versions. At the same time, the H-index of country/region, the impact factor (IF) of the journal, and the quartile of the journal category (according to the Journal Citation Reports 2020 standards) were obtained to evaluate the scientific influence of the country/region and the journal.

All the data were listed in Microsoft Excel 2016 (Redmond, WA, USA) and presented in tables or charts using Excel features. VOSviewer software (www.vosviewer.com, VOSviewer version 1.6.13) was then used to visualize the collaborative map between the countries/regions, between institutions, and between authors. The item represented in each collaboration network map was different with different research objects, but the screening condition and analysis process were the same: choose only one unit of analysis (coauthorship countries, coauthorship organizations, or coauthorship authors), and set threshold (the minimum number of documents of the studied unit was five and the maximum number of items with the greatest total link strength that could be selected was 1,000). The size of the circle of an item was proportional to its number of publications, while the width of the line between the two items was proportional to the magnitude of their collaboration. Items of the same color belonged to the same cluster, indicating that they cooperated closely in this field. The more clusters there were, the more decentralized the cooperation was. The total link strength of an item reflected the degree of cooperation with other items. The higher the value, the higher the level of cooperation. CiteSpace V (Drexel University, Philadelphia, PA, USA) is a reliable tool for mining the intellectual base and frontiers of a certain research field by performing the co-citation analysis and burst detection (13). When two references were cited by the third reference at the same time, these two references constituted a co-citation relationship. The strength of the co-citation relationship between the two cited articles was proportional to the similarity of their research contents, and this value was not invariable. The more times they were cited at the same time, the stronger the co-citation relationship was. References with strong co-citation relationships formed a certain cluster to reflect the same research topic or direction. The size of a cluster referred to the number of articles included. The silhouette value of a cluster reflected its homogeneity. The closer the value was to 1, the more homogenous it was. When the silhouette value of a cluster was >0.7, this cluster could be considered highly reliable. The nodes and links shown in the co-citation map were color-coded. Different colors represented different years. The warmer the color was, the closer the year. The nodes presented by the “tree ring” were surrounded by rings of different colors with certain thicknesses, meaning that this reference was cited in different years with a certain cited number. The color of the link between the nodes reflected the year that these two references were first co-cited. The references or keywords with the strongest citation bursts were identified to explore the most active topics in the research field. We selected the top 25 references and top 50 keywords with the strongest citation bursts in this article to explore the research hotspots in cardiac regeneration. In the results of burst detection, “begin” referred to the year the reference or keyword began to have citation burst, “end” referred to the year the reference or keyword ended the citation burst, the red line was the duration of citation burst, and “strength” referred to the intensity of its citation burst. For the setting of CiteSpace V in this study, “time slicing” was set from 2000 to 2020, while “years per slice” and “top N per slice” were set at 1 and 50, respectively (14).

To understand the structural changes of co-cited references and explore the continuously influential studies in the last 5 years, we used an alluvial diagram, and the specific principle can be seen in (15). In this study, the use of an alluvial flow map was based on the data retrieved from CiteSpace. The networks of co-cited references were initially generated in CiteSpace as Pathfinder networks of references cited by the top 50 cited articles in the last 5 years (2016–2020) and were then exported into an alluvial generator (http://www.mapequation.org/apps/AlluvialGenerator.html) directly through clicking a specific button in CiteSpace (Batch Export to Pajek.net Files). The articles presented continuously in the last 5 years were highlighted by coloring their flows (13).

## Results

### General Information

#### Growth Trend of Publications

We retrieved a total of 11,700 articles that met our screening criteria that included 8,748 articles and 2,952 reviews. The number of articles on cardiac regeneration worldwide increased yearly, from 83 in 2000 to 1,048 in 2020 (Figure 1B). Exponential, linear, logarithmic, polynomial, power, moving average, and other function types were used for trend prediction. The polynomial function was ultimately selected to fit the prediction model based on its highest *R*^2^. According to the prediction function shown in Figure 1B (y = −0.3251x^2^ + 58.434x – 34.385), ~1,094 articles related to cardiac regeneration may be published in 2021.

#### Contribution of Countries/Regions and Institutions

**Figure 2:**
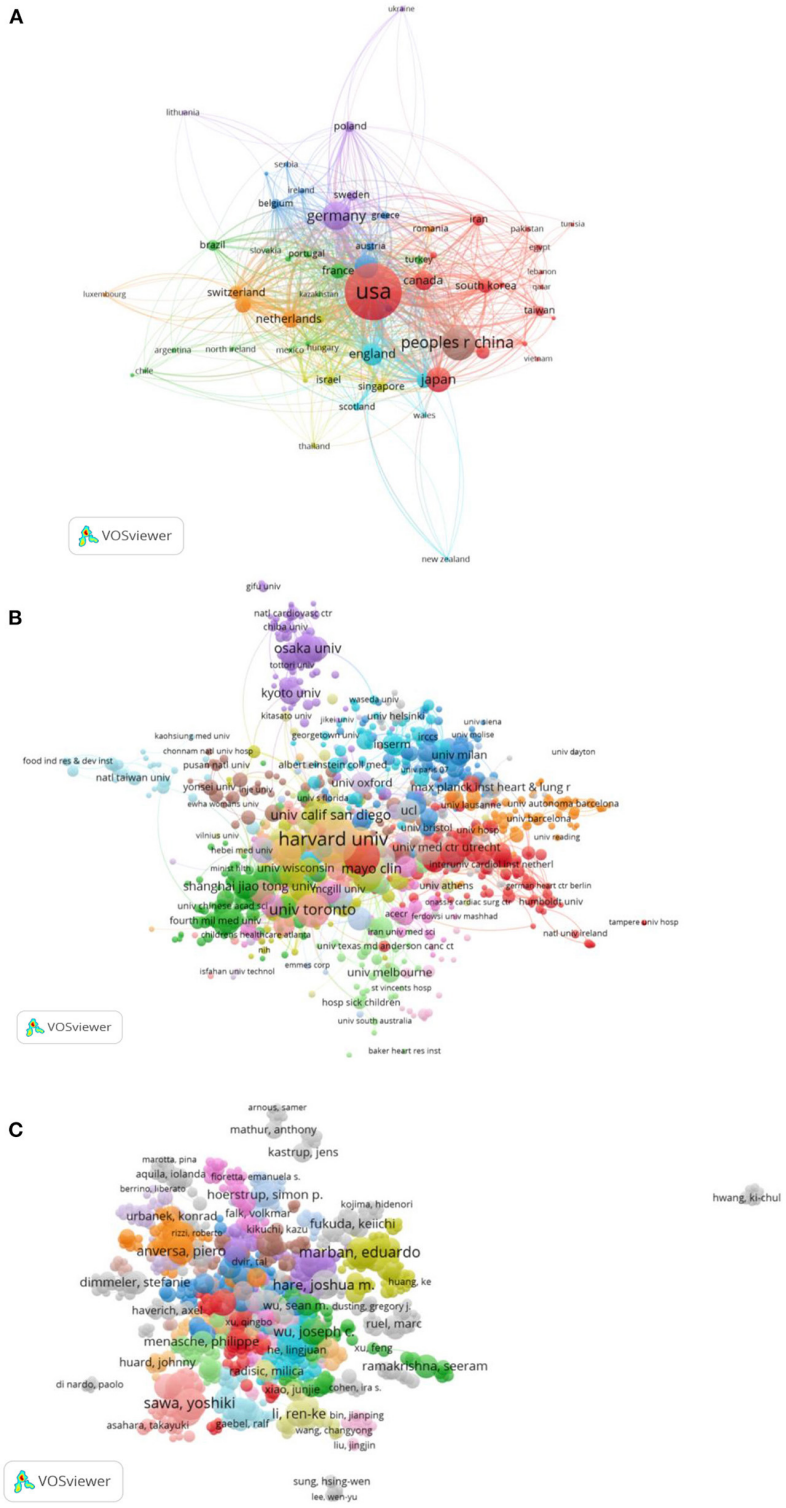
The collaboration analysis of countries/regions, institutions, and scholars in the field of cardiac regeneration. **(A)** The collaboration network of countries/regions. Eight cooperation clusters with different colors were formed with a total link strength of 5,866. **(B)** The collaboration network of institutions. Twenty-one cooperation clusters with different colors were formed with a total link strength of 17,147. **(C)** The collaboration network of scholars. Thirty-one clusters with different colors were formed with a total link strength of 21,621.

A total of 108 countries/regions and 5,936 institutions contributed to this research field, and the top 10 most productive countries/regions and institutions are listed in Tables 1, 2. The United States and Harvard University were the country and institutions with the largest number of articles, respectively, in the field of cardiac regeneration (4,627 and 522 articles), and as expected the United States ranked first in the list of productive countries/regions according to the H-index (242), indicating its highest scientific influence in this field. We then obtained and charted the annual number of articles from the top 10 productive countries/regions. As shown in Figure 1C, most of the countries/regions saw modest increases in the annual publication, while the People's Republic of China saw a rapid increase. The collaborative network maps between countries/regions and between institutions are shown in Figures 2A,B. Eight cooperation clusters between countries/regions were formed with a total link strength of 5,866, while 21 cooperation clusters between the institutions were formed with a total link strength of 17,147. From the perspective of a larger number of clusters (mixed colors), the cooperation intensity between institutions was weaker than that between countries/regions. The top 10 countries/regions and institutions that cooperated most closely with others are listed in Tables 1, 2. The United States and Harvard University had the highest total link strength (2,392 and 828), indicating their extensive cooperation with others in this research field.

**Table 1:** The top 10 countries/regions with the largest number of articles or the largest cooperation intensity.

**Rank**	**Country/region**	**Record**	**H-index**	**Rank**	**Co-authorship country/region**	**Total link strength**
1	USA	4,627	242	1	USA	2,392
2	Peoples' R China	1,614	92	2	Germany	920
3	Germany	1,177	122	3	England	773
4	Japan	892	90	4	Peoples R China	770
5	Italy	813	92	5	Italy	731
6	England	712	86	6	Netherlands	491
7	Canada	545	73	7	France	384
8	Netherlands	411	75	8	Spain	379
9	Spain	365	58	9	Australia	355
10	France	348	68	10	Japan	335

**Table 2:** The top 10 institutions with the largest number of articles or the largest cooperation intensity.

**Rank**	**Institution**	**Record**	**Rank**	**Co-authorship institution**	**Total link strength**	**Location**
1	Harvard University	522	1	Harvard University	828	USA
2	University of California System	445	2	Harvard Medical school	335	USA
3	University of London	230	3	Massachusetts General Hospital	314	USA
4	Pennsylvania Commonwealth System of Higher Education	227	4	Stanford University	304	USA
5	Brigham Women S Hospital	223	5	University Of Toronto	275	Canada
6	University of Texas System	222	6	University of California, San Diego	251	USA
7	University of Toronto	218	7	Massachusetts Institute of Technology	231	USA
8	Stanford University	206	8	Chinese Academic of Science	221	Peoples R China
9	Institut National De La Sante Et De La Recherche Medicale	196	9	Mayo Clinic	218	USA
10	University of Pittsburgh	151	10	University of California, Los Angeles	211	USA

#### Contribution of Journals

The 11,700 identified articles were published in 2,413 journals. As presented in Table 3, the number of articles published in *Circ Res* was the highest (274), followed by *PLoS ONE* (255) and *Circulation* (166). Ten of the most productive journals were classified as Q1 or Q2. All the top 10 co-cited journals, except for *PLoS ONE* (as shown in Table 3), were classified as Q1 and had a relatively higher IF (from 11.205 to 53.44). *Circulation* was the most co-cited journal with 7,465 citations, followed by *P Natl Acad Sci USA* (7,329 citations), *Circ Res* (7,308 citations), *Nature* (6,910 citations), and *Science* (5,989 citations).

**Table 3:** The top 10 productive and co-cited journals.

**Rank**	**Journal**	**Record**	**IF (2020)**	**JCR (2020)**	**Rank**	**Co-cited Journal**	**Cited time**	**IF (2020)**	**JCR (2020)**
1	Circ Res	274	17.361	Q1	1	Circulation	7,465	29.69	Q1
2	PLoS ONE	255	3.242	Q2	2	P Natl Acad Sci USA	7,329	11.203	Q1
3	Circulation	166	29.69	Q1	3	Circ Res	7,308	17.361	Q1
4	Biomaterials	158	12.473	Q1	4	Nature	6,910	49.962	Q1
5	J Mol Cell Cardiol	151	5.004	Q2	5	Science	5,989	47.728	Q1
6	Sci Rep	149	4.372	Q1	6	Cell	5,363	41.582	Q1
7	J Cell Mol Med	123	5.311	Q2	7	J Clin Invest	4,807	14.808	Q1
8	Stem Cells	122	6.277	Q1	8	PLoS ONE	4,571	3.242	Q2
9	Cardiovasc Res	110	10.787	Q1	9	Nat Med	4,565	53.443	Q1
10	Stem Cells Int	104	5.443	Q2	10	J Am CollCardiol	4,411	24.094	Q1

#### Contribution of Scholars

More than 40,000 scholars are involved in the field of cardiac regeneration. The top 10 scholars who contributed and cooperated most are presented in Table 4 separately. Anversa published the largest number of articles (84 articles) and had a strong cooperative relationship with others (the second coauthorship author with a total link strength of 415), while Wang Y also had the largest number of publications (84 articles), though his collaboration with others was relatively weak. Similar to the result of institutions, the cooperation intensity between the scholars was not as strong as that between countries/regions, reflecting in Figure 2C that 31 clusters formed with a total link strength of 21,621.

**Table 4:** The top 10 scholars with the largest number of articles or the largest cooperation intensity.

**Rank**	**Author**	**Record**	**Rank**	**Co-authorship author**	**Total link strength**
1	Anversa P	84	1	Leri A	418
2	Wang Y	84	2	Anversa P	415
3	Sawa Y	82	3	Kajsturac J	390
4	Leri A	79	4	Rota M	368
5	Marban E	76	5	Hosoda T	350
6	Kajstura J	75	6	Marban E	327
7	Shimizu T	72	7	Urbanek K	326
8	Hare JM	69	8	Sawa Y	317
9	Miyagawa S	68	9	Miyagawa S	288
10	Wang L	65	10	Cheng K	265

### Burst Detection in the Field of Cardiac Regeneration

**Figure 3:**
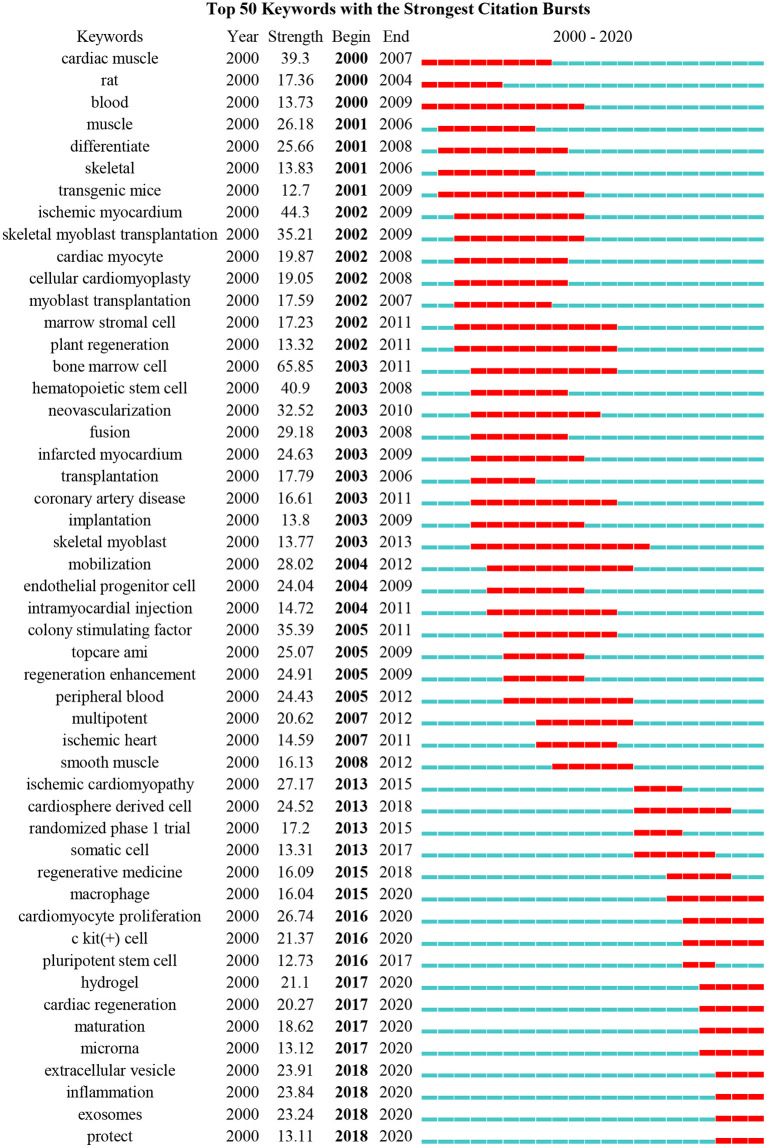
The top 50 keywords with the strongest citation bursts. Thirteen keywords, such as “Regenerative medicine,” “macrophage,” “cardiomyocyte proliferation,” “c-kit (+) cell,” “pluripotent stem cell,” “hydrogel,” “cardiac regeneration,” “maturation,” “microrna,” “extracellular vesicle,” “inflammation,” “exosomes,” and “protect,” had the citation burst since 2015.

Burst detection is a powerful tool to find and locate the hot topics increasing abruptly in a certain field. In this study, we analyzed the keywords and references with the strongest citation burst. Among the top 50 keywords with the strongest citation bursts, we mainly focused on the keywords that began to burst after the year 2015 to better understand the current research trends. Among the 13 keywords with the strongest citation burst from 2015 to 2020, cardiomyocyte proliferation, extracellular vesicle, inflammation, and exosomes were the top four keywords with the strongest burst strength (26.74, 23.91, 23.84, and 23.24, respectively) (Figure 3). In addition, “bone marrow cell,” “ischemic myocardium,” and “hematopoietic stem cell” were the top three keywords among the whole list with a burst strength of 65.85, 44.3, and 40.9, respectively.

**Figure 4:**
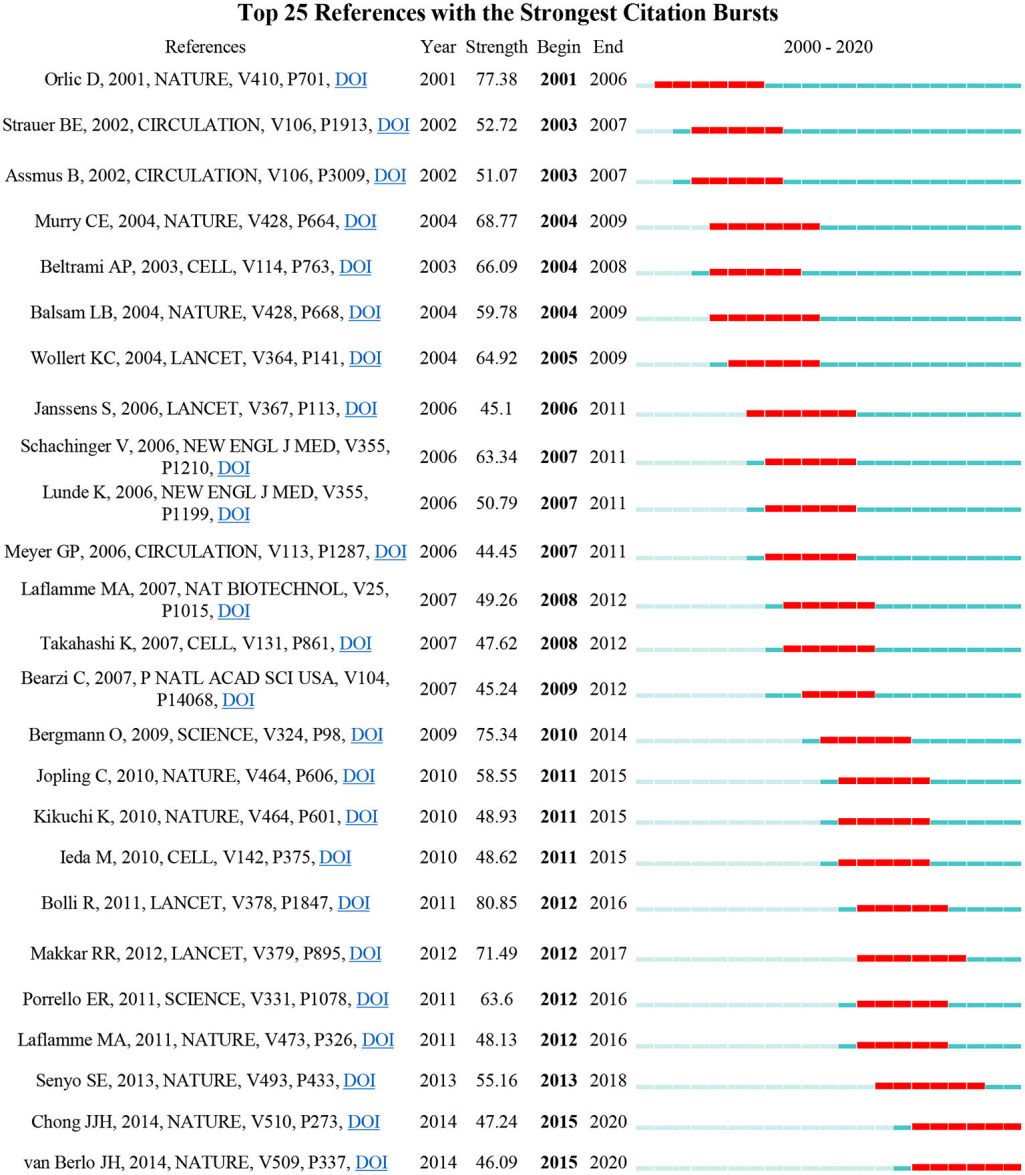
The top 25 references with the strongest citation bursts. Two articles, authored by Chong and van Berlo, began to burst after 2015.

Detection of references with the strongest citation bursts can also indicate the change of research focus in a certain area. Among the top 25 references with the strongest citation bursts, we also mainly focused on the references that began to burst after 2015 (Figure 4). Compared with the results of keywords, only two articles authored by Chong JJH and van Berlo JH were obtained with a burst strength of 47.24 and 46.09, respectively. Chong et al. demonstrated that human embryonic-stem-cell-derived CMs (hESC-CMs) could remuscularize a large number of infarcted monkey hearts, but with a potential risk of arrhythmia (16), while van Berlo indicated that c-kit-positive cells contributed only a very small percentage of CMs to the heart (17). To expand the list of influential references in recent years, we, therefore, selected the references that had a citation burst in the past 5 years. Five other articles were obtained this time, namely, the article authored by Bolli that demonstrated infusion of autologous c-kit^+^-lineage–cardiac stem cells was effective in improving the left ventricular systolic function and reducing infarct size in the patients with HF (SCIPIO trial) (18), the article authored by Porrello that proved for the first time that the mammalian heart may have the capacity to regenerate in the first few days of life (11), the article authored by Laflamme that summarized developments in the field of cardiac regeneration over the past decade and suggested that other interventions, such as adult stem cells, pluripotent stem cells, cell reprogramming, and tissue engineering may lead to better treatments or prevention of HF (19), and the article authored by Senyo SE that found that new CMs were mainly derived from the pre-existing CMs in normal mammalian myocardium homeostasis or myocardial injury (20). Interestingly, all the top 25 references with the strongest citation burst were published in the most influential journals with relatively higher IF and listed as Q1 in a certain field, such as nine in *Nature*, four in *Lancet*, three in *Circulation* or *Cell*, two in *Science* or *New Engl J Med*, and one each in *P Natl Acad Sci USA* and *Nat Biotechnol*.

### Analysis of Cited and Citing References

**Figure 5:**
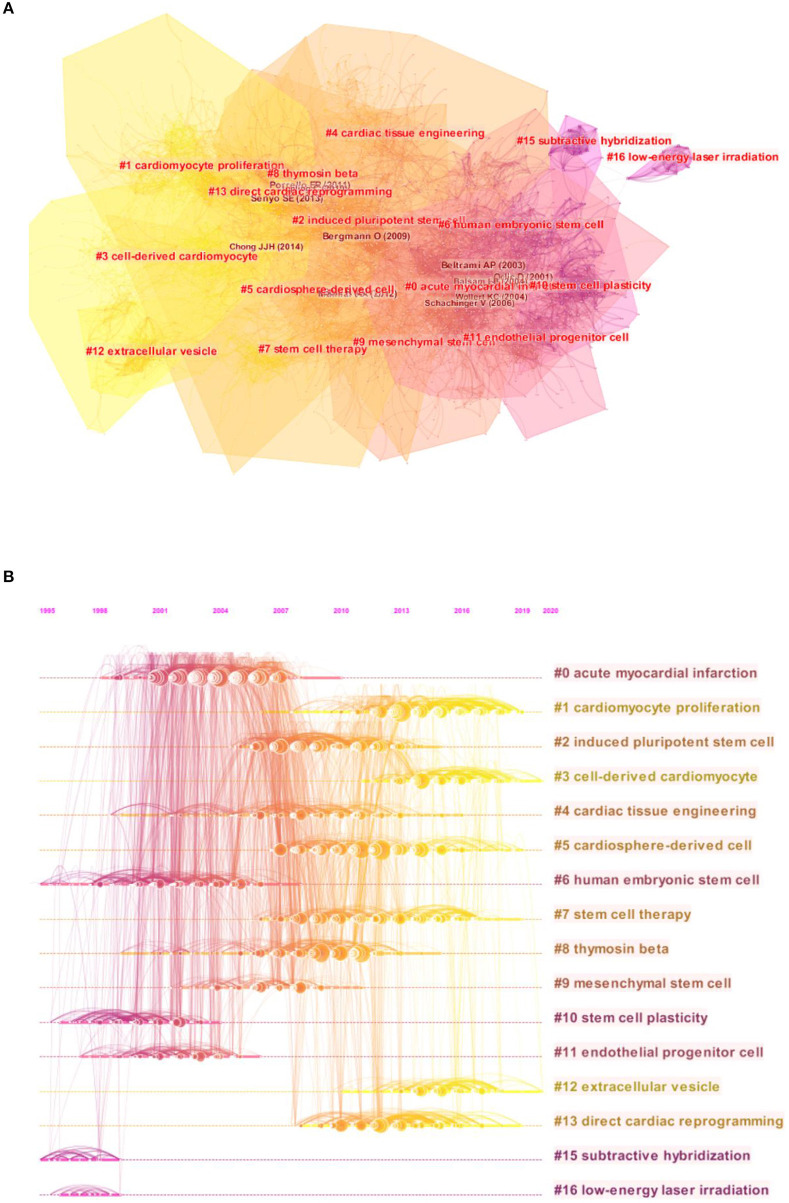
The analysis of co-citation references in the field of cardiac regeneration. **(A)** The network map of co-citation clusters. Sixteen clusters with different research topics were formed, reflecting in different colors on the map. Cluster #1 cardiomyocyte proliferation, #3 cell-derived cardiomyocyte, #5 cardiosphere-derived cell, #7 stem cell therapy, #12 extracellular vesicle, and #13 direct cardiac reprogramming were the most recent research directions. **(B)** The timeline view of co-citation clusters. Each horizontal row represented a cluster, and each node presented by a “tree ring” on the line represented a study. The line between the nodes reflected the co-citation relationship between the two studies, and the size of the node meant the number of the co-cited times.

A total of 2,510 co-cited references were obtained from the top 50 co-cited references per time slice for 2000–2020. As shown in Figure 5A, 16 clusters with different colors and sizes were formed, indicating that 16 different research topics have been concentrated in the field of cardiac regeneration in the past two decades. Figure 5B further presents the timeline changes of these clusters, revealing that clusters #1, #3, #5, #7, #12, and #13 were identified as the most recent areas. We listed the information of each cluster in Table 5. The silhouettes of 16 clusters ranged from 0.792 to 0.999, reflecting that they had relatively higher homogeneity. In addition, for clusters emerging in recent years, the number of articles in clusters #12 and #13 was relatively small, which reflected that the research in these fields was not yet mature and explained to some extent that the strength of extracellular vesicle (as reflected in the keywords “extracellular vesicle” and “exosomes”) and direct cardiac reprogramming (which did not appear in burst detection) was still lower than that of bone marrow cell and others. Moreover, Cluster #15, labeled “subtractive hybridization,” and cluster #16 labeled “low-energy laser irradiation,” had the earliest average publication year of their members (1997 and 1997, respectively), suggesting that they were earlier research topics in this field.

**Table 5:** Details of clusters.

**Cluster ID**	**Size**	**Silhouette**	**Mean (Year)**	**Label (LLR)**
0	241	0.792	2004	Acute myocardial infarction
1	163	0.896	2015	Cardiomyocyte proliferation
2	148	0.898	2009	Induced pluripotent stem cell
3	108	0.922	2015	Cell-derived cardiomyocyte
4	127	0.913	2008	Cardiac tissue engineering
5	134	0.907	2011	Cardiosphere-derived cell
6	113	0.88	2001	Human embryonic stem cell
7	127	0.929	2012	Stem cell therapy
8	109	0.922	2008	Thymosin beta
9	85	0.8786	2006	Mesenchymal stem cell
10	69	0.957	2000	Stem cell plasticity
11	79	0.936	2002	Endothelial progenitor cell
12	63	0.976	2015	Extracellular vesicle
13	58	0.975	2012	Direct cardiac reprogramming
15	10	0.992	1997	Subtractive hybridization
16	10	0.999	1997	Low-energy laser irradiation

**Figure 6:**
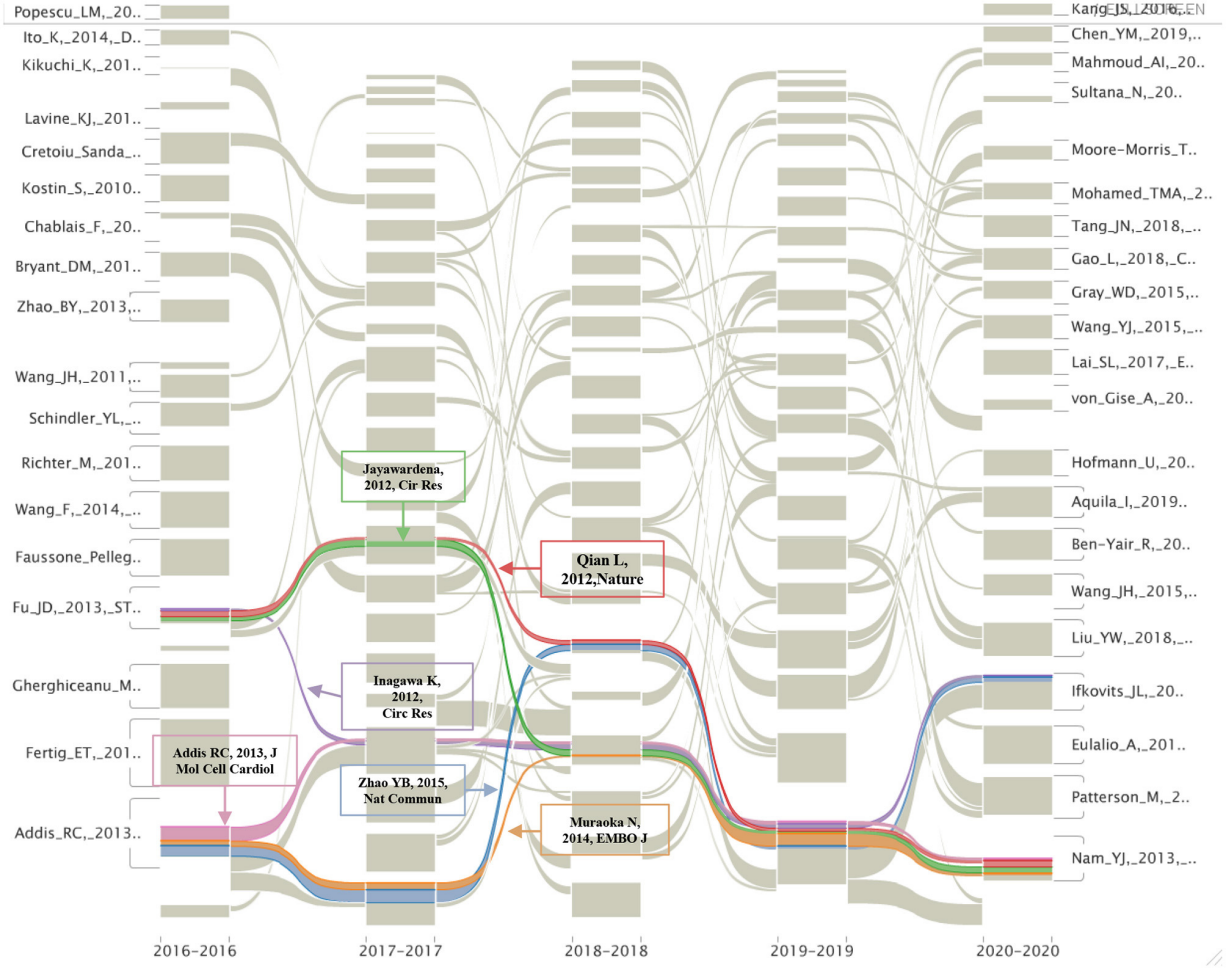
The alluvial flow map of co-cited references in the last 5 years. Each line represented a study, and colored and continuous lines referred to articles that had been consistently cited in the past 5 years.

Supplementary Table 2, lists the top five cited and citing references in clusters #1, #3, #5, #7, #12, and #13. The most cited articles in each of the above clusters were the works of Senyo, Chong, Makkar, Menasche, Ibrahim, and Qian. The alluvial diagram shown in Figure 6 represents the most cited articles in the past 5 years, and six of them (Jayawardena, 2012, *Circ Res*; Qian L, 2012, *Nature*; Inagawa K, 2012, *Circ Res*; Addis RC, 2013, *J Mol Cell Cardiol*; Zhao YB, 2015, *Nat Commun*; and Muraoka N, 2014, *EMBO J*) were cited continuously from 2016 to 2020, with three of them were related with direct cardiac reprogramming (Qian, Addis, and Zhao) and two were associated with microRNAs (miRNAs) (Jayawardena and Muraoka). Moreover, the most cited article in each of the clusters represented the emerging trend of a certain research direction. The works of Hashimoto, Mueller, Akhmedov, Banerjee, Alibhai, and Sadahiro were the most cited article in clusters #1, #3, #5, #7, #12, and #13, indicating their important contribution in the specific research direction (Supplementary Table 2).

## Discussion

With the annual death toll caused by HF increasing rapidly, finding new curative strategies is an urgent matter. Cardiac regeneration has drawn the attention of researchers since human CMs have been confirmed to have the ability to self-renew. Is manipulating adult cardiac regeneration to recover injured heart function a likely therapeutic strategy? The researchers have made a great amount of effort in the past two decades. The bibliometric analysis has made it possible to evaluate the global output of academic publications and predict future research directions of cardiac regeneration. This study provided the first bibliometric analysis of global publications on cardiac regeneration.

The increasing annual number of articles on cardiac regeneration indicates that this research field remains heated. According to the predicted function model, the number of publications in 2021 will increase to 1,094 (or ~1,094), which will be a higher number of publications in this field. Consistent with the general trend, the annual publication number of the top 10 productive countries/regions is also increasing yearly, further indicating that much work and energy is still being invested in the field of cardiac regeneration. In addition, the People's Republic of China has paid more attention to cardiac regeneration, which is closely related to the input of Chinese academic institutions (especially The Chinese Academy of Science, which is ranked eighth in the cooperation list) and the participation of Chinese scholars (Wang, who published more articles, and Cheng, who cooperated widely). However, the H-index of Chinese articles was still low, indicating that there is still much room for improvement in the quality of articles.

At the very beginning of the twenty-first century, American scholars pioneered a new era in the field of cardiac regeneration. A team at Harvard University led by Anversa et al. proposed that c-kit-positive cells may improve the possibility of cardiac regeneration. Since then, research in the field of cardiac regeneration has spread from the United States to the world (21). Over the past two decades, the United States has been the leader in this field, reflected in the highest number of published articles, the highest H-factor, four-fifths of the top 10 academic institutions by publication being located in the United States, and three-fifths of the top 10 authors by publication being American scholars (Anversa P, Leri A, Marban E, Kajstura J, Hare JM, and Terzic A). Despite the fact that c-Kit-positive cells were deposed in 2018 when members of the Harvard team, such as Anversa and Leri, were accused of academic fraud related to c-Kit cells, the United States is still leading the way in research, as evidenced by the annual number of publications, and other American scholars, such as Marban and others, are still blazing a trail in the field. In addition to the United States, the Chinese scholar Wang and Japanese scholar Sawa have made many academic achievements, but the number and influence of the published articles in China and Japan are still far behind that those in the United States. Additionally, the American institutions are still by far the most prominent contributors to this field (four-fifths of the top contributors are in the United States). Therefore, in the near future, the United States will still be the country with the largest regional advantage in the field of cardiac regeneration.

A journal is one of the marks reflecting the value of research content. According to our analysis, relevant studies were mostly published in influential journals, and those with more co-citations were often seen in the journals with world-class influence, such as *Circulation, P Natl Acad Sci USA, Circ Res, Nature, Science*, and *Cell*. These results showed that cardiac regeneration has attracted the attention of a large number of top scholars and has been a research hotspot long since and its research difficulty and value have also been recognized by scholars.

The most important information that the bibliometric analysis can provide is the intellectual base and research frontier of a certain field, which can be obtained by co-citation analysis and burst detection, respectively. Our timeline view analysis showed that the research focus in the field of cardiac regeneration has been constantly changing over the past two decades: from human embryonic stem cell (cluster #6) and stem cell plasticity (cluster #10) at the end of the twentieth century to acute myocardial infarction (cluster #0) and endothelial progenitor cell (cluster #11) at the beginning of the twenty-first century, to cardiomyocyte proliferation (cluster #1), cell-derived cardiomyocyte (cluster #3), cardiosphere-derived cell (cluster #5), stem cell therapy (cluster #7), extracellular vesicle (cluster #12), and direct cardiac reprogramming (cluster #13) in the last decade.

Among numerous research efforts on these topics, the work of Senyo and Porrello demonstrated two exciting phenomena that have influenced academia since their publication in Nature in 2013 or Science in 2011 (11, 20). Senyo et al. revealed that CMs cycle activity after normal aging and injury not only produced polyploid and multinucleate (losing the ability to proliferate) but also produced new diploid and mononuclear CMs with the ability to proliferate (the proliferation rate was ~0.76%/year) (20), while Porrello et al. indicated that the mammalian heart may have the capacity to regenerate by cardiomyocyte proliferation for a few days after birth (11). These discoveries not only initially reveal the origin of new CMs and the proliferative window of CMs after birth, but also provide a theoretical basis for exploring the factors affecting the proliferation of newborn and adult mammalian CMs, which are the landmark discoveries in the field of cardiac regeneration (reflected in the fact that they were the most cited article in cluster #1, and had relatively higher burst strength of 55.16 and 63.6). At present, elucidating the factors affecting the CMs proliferation and seeking intervention methods to improve the CMs proliferation have become key research directions (reflected in the results of burst detection and co-citation analysis). A review that published in *Nat Rev Cardiol* in 2018 and the article cited the most articles in cluster #1(cardiomyocyte proliferation), concluded that endogenous CMs proliferation could be reawakened by regulating the cell cycle regulators, the Hippo signaling pathway, and the cardiac microenvironment, but how to translate these preclinical results into clinical practice remains elusive (8). In other words, it is possible to artificially improve endogenous CMs proliferation in the theoretical and preclinical stages, but no breakthrough has yet been achieved in the clinical trials, and efforts are still needed.

In addition to enhancing endogenous CMs proliferative ability to improve cardiac regeneration, how to produce and apply exogenous CMs was widely noticed and studied. One of the basic studies in this direction was published in *Nature* by Chong et al. in 2014, the most cited article in cluster #3 (cell-derived cardiomyocyte) and one of the references with the strongest citation bursts in the past 5 years. They demonstrated that hESCs could differentiate into CMs in a large mammalian model of myocardial infarction (MI) but with a higher risk of arrhythmias (16). Consistently, Müller et al. (the article that cited the most publications in cluster #3) pointed out that despite the promising results of some preclinical trials, ESCs were always a different cell type from cardiac muscle tissue, and the proportion of transformed CMs, the maturity of transformed CMs, and the electromechanical coupling after transplantation remained challenging issues (22). In addition, although the first patient to receive hESCs-derived cardiac progenitor cells for severe HF did not develop arrhythmias and other serious complications, the large-scale clinical studies of ESCs have still been hampered, and the most obvious obstacle is ethical issues, which makes it difficult to expand the research results related to ESCs.

Cardiosphere-derived cells (CDCs) were once considered a solution to the problem of cardiac regeneration. The work of Makkar published in *Lancet* in 2012, also the most prominent study in cluster #5, proved that intracoronary infusion of autologous CDCs after MI was able to achieve the therapeutic goal of cardiac regeneration to a certain extent (23). However, some of the later studies did not observe similar results in the rats, large animals, or humans, and only modest or unnoticeable changes were found in cardiac function after injection of CDCs (10, 24, 25); therefore, the attention of CDCs has gradually decreased, and no other influential articles explaining its value in cardiac regeneration have been published in the last 5 years.

Stem cell therapy has always occupied a place in the field of cardiac regeneration, and more than 200 clinical trials have been performed to assess its efficacy (26). According to the work of Banerjee et al., which cited the most publications of cluster #7 (stem cell therapy), there are three generations of stem cells that are being explored: first-generation stem cells mainly include those derived from adult sources, especially bone marrow and adipose tissue, which are either used in unfractionated populations (e.g., bone marrow mononuclear cells [BMMNCs]) or purified cell populations [mesenchymal stem cells (MSCs)]; second-generation stem cells include progenitor cells isolated from heart tissue, human pluripotent stem cells, and cells with a specific cell phenotype that can be treated chemically or genetically; third-generation stem cells, also called the next generation, are referred to as cell combination therapy (26). As seen from the timeline map of the co-cited literature, the studies on the first and second generations of stem cells are not differentiated successively but overlap. For example, unlike the abovementioned clusters #3 and #5, which described second-generation stem cells, ESCs and CDCs, cluster #7 was mainly associated with bone marrow-derived MSCs, one of the first-generation stem cells. Two of the top five most cited articles in cluster #7 demonstrated that the allogeneic or autologous injection of MSCs was relatively safe and favorably affected the cardiac function of patients with acute myocardial infarction or ischemic cardiomyopathy (27, 28). Notably, different types of stem cells have strengths and weaknesses, and there is no evidence that anyone type is superior. At present, many stem cells have entered the stage of preclinical or clinical trials, but there is no good solution for many hindrance factors, such as ethics, tumor risk, electromechanical uncoupling, low purity and maturity of differentiated CMs, and paracrine effects, which need to be optimized in the animals and humans (26).

In addition to the above cell-based therapy, cell-free therapy is gradually moving toward the vision of scholars. In 2014, Ibrahim et al. demonstrated that the exosomes and the miRNAs they contained were the key factors in CDC-induced regeneration by proving that CDCs exosomes had the abilities to inhibit apoptosis, promote CMs regeneration, and angiogenesis (29). Based on this observation, they raised the idea that the exosomes could be used for cell-free therapy to avoid the potential risk introduced by CDCs. This work is the most cited one in the latest formed cluster #12 (extracellular vesicle), meaning it is an important foundation of this research direction. In the following years, although the number of studies on extracellular vesicle is relatively small compared with other directions (as shown in Table 5), according to the result that the keywords “extracellular microparticles” and “exosomes” are still in the stage of citation burst, it cannot be ruled out that this field will continue to flourish. In the article that cited the most in cluster #12, Alibhai et al., pointed out the inevitability and advantages of development in the field of cell-free cardiac regeneration based on the extracellular vesicles, such as exosomes, in their review and listed some unsolved problems about these nanovesicles, such as the understanding of factors contained in extracellular microparticles and their functions, how to transport these factors for cardiac regeneration or protection, and the insufficiency of clinical evidence (30). Although the extracellular microparticles still have a long way to go before they can be widely used in the clinic, the obstacles they face may be solved by biomaterials and other means; therefore, it is valuable to optimize this method, and follow-up researchers should pay more attention to this aspect.

One of the other areas that need special attention is direct cardiac reprogramming. According to co-citation analysis and alluvial flow map, direct cardiac reprogramming emerged in the last decade. In 2012, Qian et al. demonstrated that cardiac fibroblasts could be reprogrammed into induced CMs (31). This article is so influential that it has been cited continuously for the past 5 years. The work of Addis and Zhao associated with direct reprogramming also has been cited continuously. By measuring calcium function, Addis et al. demonstrated that the combination of Hand2, Nkx2.5, Gata4, Mef2c, and Tbx5 was the most effective method to realize the transdifferentiation of cardiac fibroblasts into induced CMs, while Zhao et al. emphasized that the importance of inhibiting profibrotic signaling in the process of cardiac reprogramming and using small molecules to target the transforming growth factor-β or Rho-associated kinase pathways enhanced the conversion rate up to 60% (32, 33). These discoveries emphasizing the importance of evaluation methods and multifactor regulation are great advances in the field of cardiac regeneration.

In addition to the promising directions mentioned above, it is also meaningful to study the value of macrophages, miRNAs, and inflammation in cardiac regeneration due to their constant citation burst in recent years. Macrophages are not a new topic, but their role in cardiac regeneration has always been vague due to the complex relationship between cardiac regeneration and the immune response. As described by Epelman et al. in 2015, advances in technology, such as genetic fate mapping may help to elucidate the role of macrophages in cardiac regeneration by clarifying the specific functions of the macrophage subsets and their related pathways, which also points out the direction for the research of macrophages in cardiac regeneration (34). Another review published in 2019 further clarified the research ideas on the role of macrophages in cardiac regeneration and elucidated the relationship between macrophage maturation and cardiac regeneration, together with how it became the target cell type of bioengineering exosomes to enhance the efficacy of regeneration based on clarifying the mechanism of a macrophage-dependent cardiac regenerative process (35). These works provided a relatively clear research direction that the researchers can further explore. In addition, miRNAs have potential prospects for further research. According to our results, the work authored by Jayawardena et al. and Muraoka et al. that related to miRNAs has been followed for at least 5 consecutive years, indicating their lasting scientific impact. In the study of Jayawardena et al. published in 2012, the authors first confirmed that a combination of miRNA-1,−133,−208, and−499, was able to induce direct cardiac reprogramming both *in vitro* and *in vivo*, and initially provided evidence that this type pf non-viral method may realize the goal of cardiac regeneration. However, the delivery method of miRNAs and the detailed mechanisms underlying their conversion needs to be further optimized and determined (36). Similar to the abovementioned work, Muraoka et al. found that adding miR-133 to GMT (Gata 4, Mef2, and Tbx 5) or GMTMM (Gata 4, Mef2, and Tbx 5) enhanced reprogramming by inhibiting Snai 1, and this miR-133-mediated Snai 1 inhibition was one of the key roadblocks of cardiac reprogramming (37). These studies that link miRNAs and cardiac regeneration (direct cardiac reprogramming in particular) are very attractive, as this type of small molecule is usually non-toxic. However, it should also be noted that the targets and biological functions of miRNAs are diverse, and some have not yet been clarified; therefore, the premise of safely using miRNAs in the clinic involves identifying their target molecules and pathways and maximizing the benefits of target miRNAs on cardiac regeneration, to which the researchers also need to pay attention.

The relationship between inflammation and cardiac regeneration has recently received much attention. Notwithstanding, due to the biphasic aspect of inflammation after myocardial injury, it is difficult to accurately regulate the inflammatory response to promote cardiac regeneration. Several studies found that the immune response varied with the state of regeneration, suggesting that it is possible to promote cardiac regeneration by artificially regulating the immune response (38). However, due to the sensitivity of immune cells to pathophysiological regulation, it is necessary to clarify the relationships between the immune cells (e.g., macrophages and Tregs) and between immune cells and non-immune cells before applying regulatory inflammation to the field of cardiac regeneration (38). With the development of cardiac regeneration methods, some ideas are inevitably eliminated or deemed inappropriate, the most prominent of which are c-kit-positive cells. In 2014, van Berlo et al. published an article in *Nature* and demonstrated that endogenous c-kit-positive cells may not be so important as previous findings in generating CMs (17). The publication of this study has a great impact on cardiac regeneration because it not only denies the value of c-kit cells in the field of cardiac regeneration but also confirms that cardiac regeneration cannot be easily achieved. The significance of this finding is also reflected in our analysis: this work is one of the top 25 references with the strongest citation bursts, with a durable burst from 2015 to 2020, and “c-kit (+) cell” is one of the top 50 keywords with the strongest citation bursts, with a durable burst from 2016 to 2020. Anversa et al. from Harvard University, architects of the idea that c-kit-positive cells promoted the regeneration of CMs, published several studies related to c-kit cells in recent decades, which were also largely questioned or retracted. Considering that c-kit-positive cell fraud is an important event that cannot be ignored in the development of cardiac regeneration, the results of Anversa et al. were not deleted from this bibliometric analysis. Additionally, we were aware that new directions of cardiac regeneration are emerging and believe that in the near future, c-kit cells, which have attracted much attention before, will be replaced by more reliable and effective methods. In addition, “bone marrow cell,” the keyword that had the strongest citation burst of 65.85, has gradually lost attention since 2011. In the early years, bone marrow cells represented by BMMNCs were found to enhance the left ventricular ejection fraction in several clinical trials, such as Transplantation of Progenitor Cells and Regeneration Enhancement in Acute Myocardial Infarction (TOPCARE) (39, 40), Bone Marrow Transfer to Enhance ST-Elevation Infarct Regeneration (BOOST) (41), and Finish Stem Cell study (FINCELL) (42). However, soon, the results of opposing trials appeared, such as TIME (use of adult autologous stem cells in treating people who have had a heart attack) and LateTIME (use of adult autologous stem cells in treating people 2–3 weeks after having a heart attack) that failed to observe the improvement of cardiac function and remodeling in the patients receiving BMMNCs (43, 44). The debate on bone marrow stem cells continues, and researchers are increasingly discovering the importance of purifying stem cell types to improve differentiation efficiency and develop second-generation stem cells. As the degree of stem cell purification increases (e.g., MSCs) and the number of second-generation stem cells gradually increases (e.g., ESCs and CDCs) (26), the attention given to unfractionated bone marrow stem cells has gradually decreased. There are some limitations to this study. First, we retrieved scientific outputs from WoSCC which is considered as a rigorous and reliable representation of literature and citations (13, 45); other databases, such as Google Scholar, Scopus, and PubMed, were not included. Second, we only retrieved the publications written in English; other languages were excluded, which may result in bias. Third, the information we downloaded was not the full text; therefore, some interesting details or opinions may be omitted. Nevertheless, our analysis is based on all objectively obtained information and there is no supervisor bias.

Despite these limitations, this study provides a comprehensive perspective on cardiac regeneration publications over the past 20 years. Cardiac regeneration has been a research hotspot long since, and its research value has been demonstrated by scholars. The United States and Harvard University were the leading forces in this field, while research on cardiac regeneration can be obtained from influential journals, such as *Circulation* and *Circ Res*. The research focuses in the field of cardiac regeneration has constantly changed over the past two decades: cardiomyocyte proliferation, stem cells, such as first-and second-generation stem cells, extracellular vesicles, especially exosomes, direct cardiac reprogramming, macrophages, microRNAs, and inflammation, have become research hotspots in recent years and still have great research potential and value.

## Conclusions

To our knowledge, this is the first study to use bibliometric analysis to explore the development of cardiac regeneration. The growing trend of publications on this topic indicates a mounting concern; widespread collaboration, both domestically and internationally among diverse countries, institutions, and scholars is required. Future research should focus on overcoming the limitations of the existing methods mentioned above or inventing a more applicable approach in the field of cardiac regeneration. The findings from the present study could provide insight for the researchers who are looking for research direction or guidance in their decision-making regarding cardiac regeneration and how to address HF.

## Data Availability Statement

The original contributions presented in the study are included in the article/Supplementary Material, further inquiries can be directed to the corresponding author.

## Author Contributions

SM conceived the study, carried out the data analysis, interpretation, and manuscript writing. JY helped to draft the manuscript. LC helped to collect the data. YZ and KC helped to check the data. CZ and MS helped to polish the manuscript. YL conceived the study and made final approval of the manuscript.

## Funding

This study was supported by grant from the Joint Funds of the National Natural Science Foundation of China (U1908205 to YL).

## Conflict of Interest

The authors declare that the research was conducted in the absence of any commercial or financial relationships that could be construed as a potential conflict of interest.

## Publisher's Note

All claims expressed in this article are solely those of the authors and do not necessarily represent those of their affiliated organizations, or those of the publisher, the editors and the reviewers. Any product that may be evaluated in this article, or claim that may be made by its manufacturer, is not guaranteed or endorsed by the publisher.
