# Supplementary material for: A Bibliometric and Visualized Analysis of Cardiac Regeneration Over a 20-Year Period
Source: Front Cardiovasc Med. 2021 Dec 13;8:789503. doi: 10.3389/fcvm.2021.789503 (PMC8710530; doi:10.3389/fcvm.2021.789503)
Supplement: Supplementary file 1 [file Data_Sheet_1.pdf]

Supplementary Material

Supplementary Figures

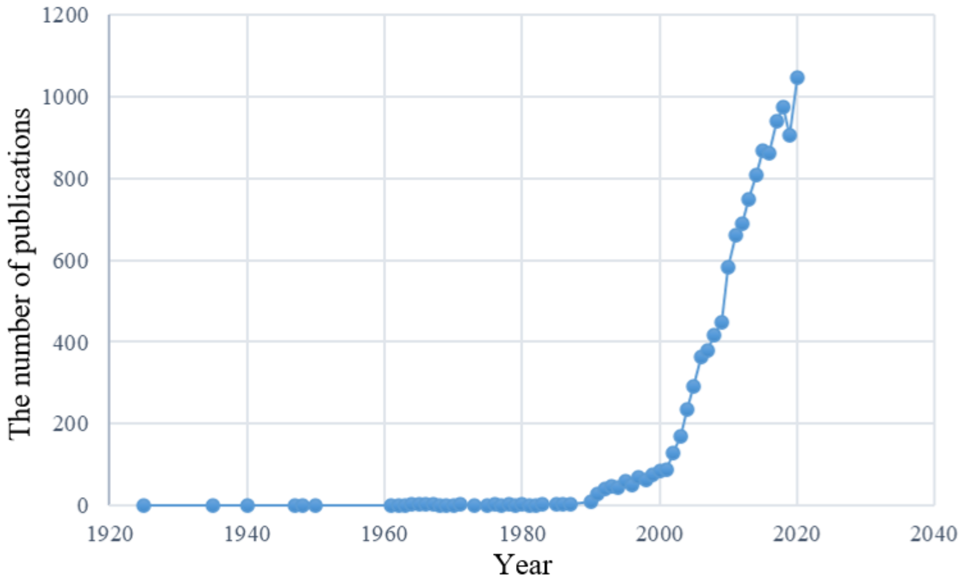

Supplementary Figure1 The annual number of publications worldwide from 1920-2020.

Supplementary Tables

Supplementary Table 1. The top 5 papers in cluster #1, 3, 5,7, 12, and 13

Cluster	Citation	Author	Publication year	Title	Journal	DOI
1	344	Senyo SE	2013	Mammalian heart renewal by pre-existing cardiomyocytes	Nature	10.1038/nature11682
1	184	Porrello ER	2013	Regulation of neonatal and adult mammalian heart regeneration by the miR-15 family.	P Natl Acad Sci Usa	10.1073/pnas.1208863110
1	184	Bergmann O	2015	Dynamics of Cell Generation and Turnover in the Human Heart.	Cell	10.1016/j.cell.2015.05.026

1	166	Duva G	2015	ERBB2 triggers mammalian heart regeneration by promoting cardiomyocyte dedifferentiation and proliferation.	Nat Cell Biol	10.1038/ncb3149
1	165	Aurora AB	2014	Macrophages are required for neonatal heart regeneration.	J Clin Invest	10.1172/JCI72181
3	280	Chong JJH	2014	Human embryonic-stem-cell-derived cardiomyocytes regenerate non-human primate hearts	Nature	10.1038/nature13233
3	118	Burridge PW	2014	Chemically defined generation of human cardiomyocytes.	Nat Methods	10.1038/nmeth.2999
3	116	Shiba Y	2016	Allogeneic transplantation of iPS cell-derived cardiomyocytes regenerates primate hearts.	Nature	10.1038/nature19815
3	116	Menasche P	2015	Human embryonic stem cell-derived cardiac progenitors for severe heart failure treatment: first clinical case report	Eur Heart J	10.1093/eurheartj/ehv189
3	113	Yang XL	2014	Engineering adolescence: maturation of human pluripotent stem cell-derived cardiomyocytes	Circ Res	10.1161/CIRCRESAHA.114.300558
5	462	Makkar RR	2012	Intracoronary cardiosphere-derived cells for heart regeneration after myocardial infarction (CADUCEUS): a prospective, randomised phase 1 trial.	Lancet	10.1038/nbt1327
5	418	Bolli R	2011	Cardiac stem cells in patients with ischaemic cardiomyopathy (SCIPIO): initial results of a randomised phase 1 trial.	Lancet	10.1016/S0140-6736(12)60195-0
5	266	Van Berlo JH	2014	c-kit+ cells minimally contribute cardiomyocytes to the heart	Nature	10.1016/S0140-6736(11)61590-0
5	220	Smith RR	2007	Regenerative potential of cardiosphere-derived cells expanded from	Circulation	10.1038/nature13309

				percutaneous endomyocardial biopsy specimens		
5	212	Bearzi C	2007	Human cardiac stem cells	P Natl Acad Sci Usa	10.1161/CIRCULATIONAHA.106.655209
7	177	Menasche P	2008	The Myoblast Autologous Grafting in Ischemic Cardiomyopathy (MAGIC) trial: first randomized placebo-controlled study of myoblast transplantation	Circulation	10.1161/CIRCULATIONAHA.107.734103
7	176	Hare JM	2012	Comparison of allogeneic vs autologous bone marrow–derived mesenchymal stem cells delivered by transendocardial injection in patients with ischemic cardiomyopathy: the POSEIDON randomized trial.	JAMA	10.1001/jama.2012.25321
7	168	Hare JM	2009	A randomized, double-blind, placebo-controlled, dose-escalation study of intravenous adult human mesenchymal stem cells (prochymal) after acute myocardial infarction.	J Am Coll Cardiol	10.1016/j.jacc.2009.06.055
7	162	Hatzistergos KE	2010	Bone marrow mesenchymal stem cells stimulate cardiac stem cell proliferation and differentiation.	Circ Res	10.1161/CIRCRESAHA.110.222703
7	146	Sanganalmath SK	2013	Cell therapy for heart failure: a comprehensive overview of experimental and clinical studies, current challenges, and future directions	Circ Res	10.1161/CIRCRESAHA.113.300219
12	145	Ibrahim AGE	2014	Exosomes as critical agents of cardiac regeneration triggered by cell therapy	Stem Cell Reports	10.1016/j.stemcr.2014.04.006
12	115	Khan M	2015	Embryonic stem cell-derived exosomes promote endogenous repair mechanisms and enhance cardiac function following myocardial infarction	Circ Res	10.1161/CIRCRESAHA.117.305990
12	113	Barile L	2014	Extracellular vesicles from human cardiac progenitor cells inhibit cardiomyocyte apoptosis and improve	Cardiovasc Res	10.1093/cvr

				cardiac function after myocardial infarction		
12	84	Gallet R	2017	Exosomes secreted by cardiosphere-derived cells reduce scarring, attenuate adverse remodelling, and improve function in acute and chronic porcine myocardial infarction	Eur Heart J	10.1093/eurheartj/ehw240
12	69	Gray WD	2015	Identification of therapeutic covariant microRNA clusters in hypoxia-treated cardiac progenitor cell exosomes using systems biology	Circ Res	10.1161/CIRCRESAHA.116.304360
13	268	268	2012	In vivo reprogramming of murine cardiac fibroblasts into induced cardiomyocytes	Nature	10.1038/nature11044
13	252	252	2010	Direct reprogramming of fibroblasts into functional cardiomyocytes by defined factors	Cell	10.1016/j.cell.2010.07.002
13	238	238	2012	Heart repair by reprogramming non-myocytes with cardiac transcription factors	Nature	10.1038/nature11139
13	176	176	2012	MicroRNA-mediated in vitro and in vivo direct reprogramming of cardiac fibroblasts to cardiomyocytes	Circ Res	10.1161/CIRCRESAHA.112.269035
13	122	122	2013	Reprogramming of human fibroblasts toward a cardiac fate	J Am Coll Cardiol	10.1016/j.jacc.2011.05.013

Supplementary Table 2. The top 5 papers that cited the members of cluster #1, 3, 5, 7, 12, 13

Cluster	Coverage	Author	Year	Citing paper title	Journal	DOI
1	49	Hashimoto H	2018	Therapeutic approaches for cardiac regeneration and repair.	Nat Rev Cardiol	10.1038/s41569-018-0036-6
1	47	Foglia MJ	2016	Building and re-building the heart by cardiomyocyte proliferation.	Development	10.1242/dev.132910
1	46	Lin ZQ	2014	Strategies for cardiac regeneration and repair	Sci Trans med	10.1126/scitranslmed.3006681
1	46	Galdos FX	2017	Cardiac regeneration lessons from development	Circ Res	10.1161/CIRCRESAHA.116.309040
1	45	Karra R	2017	Redirecting cardiac growth mechanisms for therapeutic regeneration.	J Clin Invest	10.1172/JCI89786
3	51	Mueller P	2018	Stem cell therapy in heart diseases-cell types, mechanism and improvement strategies	Cell Physiol Biochem	10.1159/000492704
3	37	Ebert AD	2015	Reprogramming and transdifferentiation for cardiovascular development and regenerative medicine where do we stand?	EMBO Mol Med	10.15252/emmm,201504395
3	36	Banerjee MN	2018	Clinical studies of cell therapy in cardiovascular medicine recent development and future direction	Circ Res	10.1161/CIRCRESAHA.118.311217

3	32	Montero P	2020	Cell, materials, and fabrication processes for cardiac tissue engineering	Front Bioeng Biotech	10.3389/fbioe.2020.00955
3	31	Barreto S	2019	Cardiac progenitor cells from stem cells: learning from genetics and biomaterials	Cells	10.3390/cells8121536
5	51	Akhmedov AT	2013	Myocardial regeneration of the failing heart	Heart Fail Rev	10.1007/s10741-012-9348-5
5	44	Davis DR	2011	Autologous cell therapy for cardiac repair	Expert Opin Biol Ther	10.1517/14712598.2011.556615
5	39	Kikuchi K	2012	Cardiac regenerative capacity and mechanisms	Annu Rev Cell Dev Biol	10.1146/annurev-cellbio-101011-155739
5	36	Malliaras K	2011	Cardiac cell therapy where we've been, where we are, and where we should be headed	Br Med Bull	10.1093/bmb/ldr018
5	35	Doppler SA	2013	Cardiac regeneration: current therapies-future concepts	J Thorac Dis	10.3978/j.issn.2072-1439.2013.08.71
7	32	Banerjee MN	2018	Clinical studies of cell therapy in cardiovascular medicine recent development and future direction	Circ Res	10.1161/CIRCRESAHA.118.311217
7	31	Akhmedov AT	2013	Myocardial regeneration of the failing heart	Heart Fail Rev	10.1007/s10741-012-9348-5
7	28	Ghiroldi A	2017	Regenrating the human heart: direct reprogramming strategies and their current limitation	Basic Res Cardiol	10.1007/s00395-017-0655-9
7	28	Di salvo TG	2015	Evolving targeted therapies for right ventricular failure	Expert Opin Biol Ther	10.1517/14712598.2015.1054277
7	27	Davis DR	2011	Autologous cell therapy for cardiac repair	Expert Opin Biol Ther	10.1517/14712598.2011.556615
12	24	Alibhai FJ	2018	Emerging role of extracellular vesicles in cardiac repair and rejuvenation	Am J Physiol Heart Circ Physiol	10.1152/ajpheart.00100.2018

12	21	Bollini S	2018	Triggering endogenous cardiac repair and regeneration via extracellular vesicle-mediated communication	Front Physiol	10.3389/fphys.2018.01497
12	19	Yuan Y	2018	Stem cell-derived exosome in cardiovascular diseases: macro roles of micro particles	Front Pharmacol	10.3389/fphar.2018.00547
12	18	Bei Y	2017	Extracellular vesicles in cardiovascular theranostics	Theranostics	10.7150/thno.21274
12	18	Dougherty JA	2017	Potential role of exosomes in mending a broken heart nanoshuttles propelling future clinical therapeutics forward	Stem Cells Int	10.1155/2017/5785436
13	27	Sadahiro T	2015	Direct cardiac reprogramming progress and challenges in basic biology and clinical applications	Circ Res	10.1161/CIRCRESAHA.116.305374
13	27	Ebrahimi B	2017	In vivo reprogramming for heart regeneration: a glance at efficiency, environmental impacts, challenges and future directions	J Mol Cell Cardiol	10.1016/j.yjmcc.2017.05.005
13	26	Kojima H	2017	Discovery and progress of direct cardiac reprogramming	Cell Mol Life Sci	10.1007/s00018-017-2466-4
13	24	Ghiroldi A	2017	Regenerating the human heart direct cardiac reprogramming strategies and their current limitations	Basic Res Cardiol	10.1007/s00395-017-0655-9
13	23	Haginiwa S	2017	Direct cardiac reprogramming	Cardiac regeneration	10.1007/978-3-319-56106-6_6
13	23	Doppler SA	2015	Direct cardiac reprogramming-the future of cardiac regeneration	Int J Mol Sci	10.3390/ijms160817368
13	23	Batty JA	2017	Direct cellular reprogramming for cardiac repair and regeneration	Eur J Heart Fail	10.1002/ejhf.446
